# Facilitation of team-based care to improve HTN management and outcomes: a protocol for a randomized stepped wedge trial

**DOI:** 10.1186/s12913-023-09533-1

**Published:** 2023-05-31

**Authors:** Donna R. Shelley, Dominique Brown, Charles M. Cleland, Hang Pham-Singer, Dina Zein, Ji Eun Chang, Winfred Y. Wu

**Affiliations:** 1 New York University School of Global Public Health, New York, NY USA; 2 NYU Grossman School of Medicine, New York, NY USA; 3 New York City Department of Health and Mental Hygiene, Long Island City, NY USA; 4 University of Miami Miller School of Medicine, Miami, FL USA

**Keywords:** Team-based care, Practice facilitation, Implementation, Hypertension, Cardiovascular diseases, Primary care

## Abstract

**Background:**

There are well-established guidelines for treating hypertension (HTN), yet only half of patients with HTN meet the defined target of < 140/90. Team-based care (TBC) is an evidence-based strategy for improving blood pressure (BP) management and control. TBC is defined as the provision of health services by at least two health professionals “who work collaboratively with patients and their caregivers to accomplish shared goals to achieve coordinated, high-quality care”. However, primary care practices experience challenges to implementing TBC principles and care processes; these are more pronounced in small independent practice settings (SIPs). Practice facilitation (PF) is an implementation strategy that may overcome barriers to adopting evidence-based TBC to improve HTN management in SIPs.

**Methods:**

Using a stepped wedge randomized controlled trial design, we will test the effect of PF on the adoption of TBC to improve HTN management in small practices (< 5 FTE clinicians) in New York City, and the impact on BP control compared with usual care. We will enroll 90 SIPs and randomize them into one of three 12-month intervention waves. Practice facilitators will support SIPs to adopt TBC principles to improve implementation of five HTN management strategies (i.e., panel management, population health, measuring BP, supporting medication adherence, self-management). The primary outcome is the adoption of TBC for HTN management measured at baseline and 12 months. Secondary outcomes include the rate of BP control and sustainability of TBC and BP outcomes at 18 months. Aggregated data on BP measures are collected every 6 months in all clusters so that each cluster provides data points in both the control and intervention conditions. Using a mixed methods approach, we will also explore factors that influence the effectiveness of PF at the organization and team level.

**Discussion:**

This study will provide much-needed guidance on how to optimize adoption and sustainability of TBC in independent primary care settings to reduce the burden of disease related to suboptimal BP control and advance understanding of how facilitation works to improve implementation of evidence-based interventions.

**Trial registration:**

ClinicalTrials.gov; NCT05413252.

**Supplementary Information:**

The online version contains supplementary material available at 10.1186/s12913-023-09533-1.

## Background

Hypertension (HTN) accounts for nearly 400,000 preventable CVD-related deaths per year in the U.S. [1, 2]. Team-based care (TBC), defined as the provision of health services by at least two health professionals “who work collaboratively with patients and their caregivers to accomplish shared goals to achieve coordinated, high-quality care” [3, (Naylor MD, Coburn KD, Kurtzman ET, et. al.: Inter-professional team-based primary care for chronically ill adults: state of the science, unpublished)] is an evidence-based strategy for improving BP management and control [4, 5]. Two systematic reviews examining 80 studies, found that TBC resulted in a median improvement of the proportion of patients with BP control (defined as BP < 140/90) of 12.0% [4, 5]. Another review of 35 studies that focused on patients with diabetes mellitus found similar improvements in BP control [6]. The strength of the evidence underpins the endorsement of TBC by the consensus American College of Cardiology and American Heart Association Task Force in its latest BP management guidelines [7, 8].

Despite the effectiveness of TBC, primary care practices experience significant barriers to implementing TBC care processes [9–11]. For example, a core component of TBC is the allocation of specific care management roles to non-physician members of the team (e.g., medical assistants who assess patients’ medication adherence). However, prior studies have found that cultural silos within practices perpetuate beliefs that non-physician staff cannot expand their roles [12, 13]. Other barriers include lack of experience and local expertise in TBC, insufficient quality improvement (QI) infrastructure to support TBC approaches that are aligned with existing BP management guidelines (e.g., population health systems), and perceived lack of reimbursement for TBC processes [10, 11, 14–16].

Barriers to implementing TBC are more pronounced in small independent primary care practices (SIPs) [17]. SIPs (< 10 clinicians) are significantly less likely than larger practices and those owned by hospital systems to adopt and use critical TBC-related care structures and processes for QI, including HTN care management facilitated by health information technology (e.g., registries, medication adherence management) [17]. This gap is due to a comparative lack of resources and staff expertise in information systems and practice redesign that translates into less capacity for adopting TBC [17, 18]. There is a strong rationale for studying strategies for implementing TBC in SIPs. Despite recent trends toward the acquisition and consolidation of SIPs into larger healthcare systems in the U.S., these practices continue to play a major role in U.S. healthcare delivery; more than half of primary care visits occur in small practice settings [19–21].

Our experience conducting research with SIPs has demonstrated the need for external support such as practice facilitation (PF) to scale the adoption of evidence-based practice models like TBC [18, 22–26]. PF is a multifaceted implementation strategy for supporting primary care practices’ efforts to improve clinical practice quality [27–29] and adherence to evidence [30] and has the potential to address challenges to adopting TBC to improve HTN management in small primary care settings. Stetler and colleagues define facilitation as the “deliberate and valued process of interactive problem-solving and support [31].” More recently, Berta and colleagues provided a theoretically-grounded definition of facilitation that places practice context at the center: “Facilitation is a goal-oriented, context-dependent social process for implementing new knowledge into practice or organizational routines [32].” Facilitators build practice capacity using a range of organizational development and practice improvement approaches including stakeholder engagement, training, clarifying roles and responsibilities, supporting goal setting, providing technical assistance to optimize data systems and reporting for ongoing monitoring and evaluation, and managing team processes. This study will evaluate the impact of PF on the adoption of TBC principles for HTN management and BP outcomes in small practices that are members of a primary care practice network in New York City.

## Methods

### Study setting

All enrolled practices are members of the New York City (NYC) Department of Health and Mental Hygiene (DOHMH)’s NYC Regional Electronic Adoption Center for Health (REACH) Network. The Network is managed by the DOHMH Bureau of Equitable Health Systems (BEHS). NYC REACH supports over 2,000 small- and medium-sized independent practices with a range of services to enhance infrastructure (e.g., support EHR updates) and optimize care processes to improve health outcomes. Overwhelmingly, participating practices are in socioeconomically disadvantaged, racially diverse neighborhoods with evidence of significant disparities in HTN control and CVD-related outcomes more generally [33–38].

### Study design

Using a stepped wedge randomized controlled trial (RCT) design, we randomly assign 90 practices to one of three waves (Table 1). Waves are separated by 6 months (i.e., a new wave enters the intervention phase every 6 months), and the intervention period lasts 12 months. Data collection for the primary outcome (i.e., adoption of TBC) occurs immediately prior to the beginning of the intervention period and at 12 months. BP control (i.e., secondary outcome) is measured every six months in all clusters at every time period so that each cluster provides data points in both the control and intervention conditions.

**Table 1:**
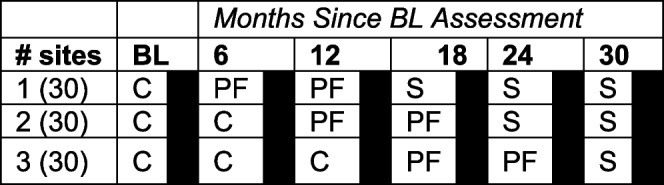
Stepped-wedge study design. (  =TBC data collection) *BL *Baseline period, *C *Control, *PF *Practice facilitation period, *S *Sustainability

### Conceptual framework

The study design and assessments are informed by principles of TBC, and Armenakis’s theory of organizational readiness [39]. Armenakis’s theory posits that successful implementation of a change effort is determined by the extent to which the change recipient perceives a need for change, is confident in their ability to change, believes there is commitment from formal and opinion leaders, believes a change will be beneficial, and believes that the change is appropriate.

**Fig. 1:**
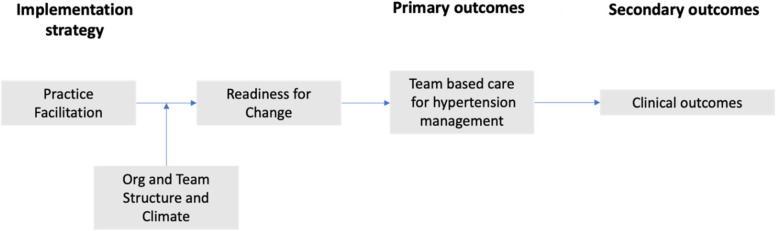
HHTeams conceptual framework

We hypothesize that additional organizational features (e.g., staffing, resources) will moderate the effectiveness of PF (Fig. 1). We further hypothesize that PF will increase the adoption of TBC principles and HTN management strategies by increasing organizational readiness (e.g., clinician efficacy and motivation to implement the practice changes necessary to improve HTN outcomes) [39, 40]. Qualitative assessment of the contextual barriers to and facilitators of implementing TBC for improving HTN management will be further guided by the Consolidated Framework for Implementation Research (CFIR) [41]. The CFIR broadens the inquiry by capturing additional organizational, individual, and intervention characteristics that may impact implementation effectiveness and sustainability and the process by which practice change is achieved, including the role of practice facilitators as change agents.

### Practice eligibility

Eligible practices must have fewer than 5 full-time equivalent (FTE) primary care providers – MD, DO, NP, PA – providing primary care), a minimum of two staff (e.g., medical assistant, front office staff, nurse), minimum of 200 patients in the practice that have a diagnosis of HTN, and less than 75% of patient with HTN have a BP < 140/90 in the past 6 months.

### Practice recruitment and retention

The study recruits eligible practices from the NYC REACH network. First, NYC REACH recruitment staff uses their existing database to pre-screen practices based on the eligibility criteria. Next, recruitment staff reach out by phone to the lead clinicians to describe the study and assess interest. If interested, they are asked to respond to survey questions that confirm eligibility. If eligible, lead clinicians are asked to sign a memorandum of understanding (MOU) which outlines expectations and requirements for participation. BEHS uses several approaches to retain practices once all sites are recruited and randomized. For practices waiting for their wave to begin the intervention, BEHS’ primary strategy is to stay in regular contact through a series of activities that were not related to the project. This includes their usual modes of communication to keep practices up-to-date on relevant changes in health care policies and to provide trainings and staff orientations, again, on topics that are not related to the project’s main aim. Practice facilitators made contact by email, phone, and/or in person 1 month prior to the start of the subsequent study waves.

### Intervention conditions

#### Usual care

During the usual care period, patients at the sites receive standard HTN care delivered by their primary care providers.

#### Practice facilitation

Facilitators collaborate with practice leadership and staff to implement TBC principles (i.e., shared goals, clear roles and responsibilities, effective communication, mutual trust and measurable processes/outcomes (i.e., monitoring and evaluation)) to facilitate the adoption of five evidence-based HTN management strategies [11, 42, 43]. These include: 1) population health (e.g., collecting patient race and ethnicity data to address disparities), 2) panel management (e.g., identifying and reaching out to patients who have uncontrolled HTN), 3) measuring BP accurately, 4) improving medication adherence, and 5) self-management support. Facilitators use a toolkit, developed by BEHS and the research team, that describes the activities to be completed before, during, and after each visit. Facilitators are expected to complete at least 16 in-person practice site visits during the 12-month intervention (Table 2). The purpose of the first two visits is to orient practices to the goals of the project and to conduct a needs assessment. This includes evaluating current roles and responsibilities for each member of the practice and obtaining a baseline assessment of care processes. For example, assessing the current application of TBC principles, in relation to HTN management (e.g., whose role is it to assess BP, what are their modes of communication).

**Table 2:**
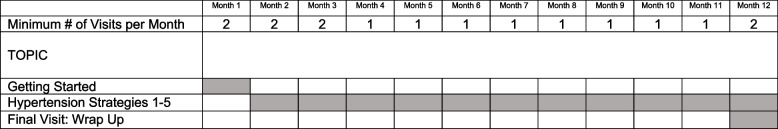
12-Month Practice Facilitation Intervention

#### Practice facilitation visits

In visit 1, facilitators use an adapted version of the Primary Care Team Guide Assessment (PCTGA) [44] to establish current workflows across key domains related to TBC. Specifically, the tool was adapted to assess at baseline the extent to which practices have adopted principles of TBC in the context of using specific HTN management strategies. For example, the tool assesses to what extent the practice defines team roles and practice goals in relation to providing self-management support. For each domain, practices are scored along a continuum from level D (just beginning to make changes) to level A (has achieved the most important changes in that domain). Practice facilitators are also trained to use the tool as a method for engaging practices in a discussion about expected challenges to integrating a TBC approach into current workflows, and specifically, to implement evidence-based HTN strategies. Using findings from the PCTGA and the roles and responsibilities worksheet, the facilitators complete a HTN management checklist after the first visit. These three tools provide the necessary information to support practice facilitators in tailoring the intervention to practice context. During the second visit, facilitators use their findings to further engage the practice in exploring potential challenges to implementing TBC and HTN strategies, review the practice’s baseline BP quality measures, and begin to expand on core QI concepts (e.g., plan do study act cycles and performance reporting). In the following visits, facilitators work with practices to integrate a TBC approach for each of the five HTN management strategies. Table 3 outlines the PF activities for those visits. These include conducting workflow analyses, using root cause analyses to identify barriers and suggest tailored solutions, and supporting the practice to redefine roles and responsibilities based on those analyses and updating electronic health record (EHR) templates. For the final visit, facilitators reassess practice progress using the PCTGA and reinforce action steps for sustaining gains.

**Table 3:** Overview of activities by visit for the 12-month PF intervention

Visits	Activities
Web-based “kick-off” learning session	● Each wave of practices attends a virtual meeting to review the purpose of the study, the evaluation plan (e.g. surveys), and discuss team-based care and the application to improving HTN management and outcomes
Getting Started Part 1 (Visit 1)	● Provide overview of the principles of TBC, the five HTN strategies, and an explanation of the benefits of TBC for HTN management ● Meet with team members to assess roles and responsibilities and conduct PCTGA needs assessment ● Meet with practice staff to assess general roles and responsibilities
Getting Started Part 2 (Visit 2)	● Introduce how SIPs can implement the HTN strategies using TBC ● Explore potential challenges to using TBC to implement the HTN strategies in a small practice ● Review the practice’s BP control rate ● Introduce and define the concept of QI ● Set expectations for working on this QI project ● Help establish routine communication channels among the team ● Introduce Root Cause Analysis (RCA) ● Lead the practice care team through an RCA exercise
HTN Strategies 1—5	● Obtain and review monthly HTN control rate and any other relevant quality measure specific for the strategy ● Review/Discuss the relevance and importance for supporting the strategy ● Conduct workflow mapping of how the practice currently implements the strategy ● Assess barriers to implementing the strategy ● Conduct an RCA of identified barriers to implementing the strategy ● Assess whether the EHR is set-up to support to support the strategy ● Help set-up the EHR to support the strategy (if necessary) ● Determine if the practice care team is appropriately using the EHR to support the strategy ● Determine training needs to support the strategy ● Describe/review/provide trainings for the best practices for implementing the strategy ● Discuss how TBC elements can be integrated into the workflow to support the strategy ● Agree on a clear shared practice goal for the strategy ● Support the practice care team to develop a new workflow ● Support the practice care team to implement an PDSA cycle to integrate new workflow and assess practice goal (outcome) ● Evaluate the PDSA cycle and revise workflow as needed
Wrap up	● Review the 6-month BP control rate ● Review the 6-month race/ethnicity documentation rate ● Conduct the PCTGA ● List gains the practice care team has made ● Confirm which new workflows the practice care team will sustain after the project ● Establish actionable steps the practice care team can take to continue improvements and sustain gains ● Confirm which established routine communication channels the team will continue to use to enable sustainability ● Complete the HTN Management Checklist

*Abbreviations EHR* Electronic Health Record, *HTN* Hypertension *PCTGA* Primary Care Team Guide Assessment, *QI* Quality Improvement, *RCA* Root Cause Analysis, *TBC* Team-based Care

#### PF Training

Practice facilitators attend 15 h of didactic and experiential training over three days. BEHS facilitators are all certified facilitators and have received additional training on motivational interviewing, and implementing quality improvement strategies and systems (e.g., adapting the practice EHR to generate population-based blood pressure reports). Therefore, the training focuses on increasing capacity to support practices to apply principles of TBC in the context of improving the implementation of the five HTN management strategies. Specifically, the training reviews TBC principles, HTN management strategies, the activities that they are expected to complete during each visit, including conducting workflow and RCA, and uses case examples to engage facilitators in role-playing exercises. Practice facilitators are also trained to complete the assessment tools. Prior to attending the trainings, facilitators are expected to review the PF toolkit, complete required readings, and view required videos. The required readings focus on teams, team functioning, and the principles of TBC, while the videos focus on leadership, communication, and fostering mutual trust and support in primary care practices.

#### PF Supervision

Facilitators participate in weekly one-on-one meetings with a PF supervisor. Supervision meetings review site visits and facilitators/barriers to implementing TBC and HTN management strategies. Supervisors use the PCTGA and other tools that the PFs completed, as well as PF visit data from Salesforce, a cloud-based customer relations management system [45], (see fidelity measures, below) to guide the conversation. PF supervisors use a standardized form to guide the discussion and document challenges and the next steps to address those challenges. Additionally, facilitators meet monthly to share field experiences. Based on supervision meetings, additional PF training is provided as necessary.

#### Measures and data collection

A secure, online, password-protected database built on a REDCap platform and linked to a secure web-based platform is used for survey collection and tracking. All EHR data is aggregated count data only. These data after extraction from the EHR are exchanged via secure file transfer protocol between BHES and NYU. The transfer of deidentified salesforce data follows the same data security protocol. Interviews are audiotaped, transcribed and stored in a password protected database. An independent data monitoring safety board is convened annually to review study progress, and monitor study participant safety.

#### Primary outcome

To measure our primary outcome (Table 4), adoption of TBC for improving adoption of HTN strategies, we adapted two instruments: the 29-item Primary Team Dynamics Survey Measure and a subscale from the Team Process Survey Measure. The Primary Team Dynamics Survey Measure is a validated instrument designed to measure team dynamics in primary care settings [46]. The 29-item instrument examines seven factors that map to four out of five elements of TBC: clear roles, shared goals, mutual trust, and effective communication. Respondents will be asked to rate their level of agreement on a scale from 1 (*Strongly disagree*) to 5 (*Strongly agree*) to statements related to TBC. Participants also complete the 5-item monitoring progress toward goals subscale of the team process measure. Respondents rate the extent to which their team participates in activities related to goal monitoring on a scale of 1 (*Not at all*) to 5 (*To a very great extent*). Practice staff in each wave complete the TBC Assessment Tool at baseline and 12- and 18-month period (end of intervention period). Both surveys were adapted to include language specifically related to HTN management. This allows us to measure the degree to which practice teams adopt TBC strategies for HTN management.

**Table 4:** Measures and data source

	MEASURES	DATA SOURCE/COLLECTION
Primary Outcome	Adoption of team-based care for improving adoption of hypertension strategies (TBC) [46]	Provider and staff survey Baseline & 12 months
Secondary Outcomes:	Clinical outcome: BP control defined as proportion of patients with HTN who achieve BP < 140/90 (NCQA HEDIS)	EHR every 6 months for 12 months
Implementations outcomes	Fidelity: Practice facilitation [47]	Salesforce tracking system PF interviews
	Sustainability of TBC for implementing HTN management strategies and BP outcome	Provider and staff survey at 18 months EHR data at 18 months
Practice Context	Organizational readiness [39] Barriers and facilitators Staffing, financial resources Staff and provider burnout and job satisfaction	Provider and staff survey baseline and 12 months Qualitative interviews 12 months

#### Secondary outcome

We assess the percentage of patients with BP < 140/90 [48]. Data on BP is obtained through the EHR. Using the National Quality Forum-endorsed BP control measure [48], the denominator is defined as the count of all individuals, ages 18–85, with a diagnosis of HTN, who were seen at least once during the measurement period. The numerator represents the count of individuals who met the denominator criteria whose latest measured BP in the measurement period was less than 140/90 [49]. We obtain reports on BP control at baseline, and every six months thereafter, coinciding with the stepped-wedge design, using the most recent prior 6-month measurement period. BP data is extracted from the EHR systems through existing reporting and registry tools. We also collect aggregated data on race/ethnicity and insurance status among patients with HTN.

#### Implementation outcomes

##### Fidelity

Facilitators are expected to complete activities outlined in the toolkit before, during, and after each visit in the 12-month intervention and document these activities using Salesforce. Practice facilitators complete a visit checklist that reflects items outlined in Table 2 and is embedded in Salesforce. This system provides an easily accessible and user-friendly system for facilitators to document their work and for supervisors and the investigative team to assess adherence to the protocol and the degree to which components of the PF strategy are delivered, with the understanding that activities are tailored to the practice setting. Practice facilitators are also asked to document how much time was allotted to completing pre-visit, visit and post-visit activities. Finally, facilitators use Salesforce to document activities conducted in between visits such as follow-up communication with practices (sent email and phone calls).

##### Sustainability

Sustainability of the primary (i.e., TBC principles to implement HTN strategies) and secondary outcomes (i.e., BP control) will be assessed at 18 months, six months after the intervention period ends.

#### Practice context

##### Provider/Staff characteristics and satisfaction

Practice staff are asked to complete a survey at baseline which assess practice and staff level characteristics, and organizational readiness for change. The study collects staff-level sociodemographic data including role, race, ethnicity, sex, age, country of birth, and spoken languages. Practice-level characteristics include practice location, ownership, number of staff, and NCQA Patient-Centered Medical Home recognition.

All staff are asked to answer the statement “Overall, I am satisfied with my current job” and rate their satisfaction from 1 (*Strongly disagree*) to 5 (*Strongly agree*) [50]. Additionally, all staff are asked “Using your own definition of ‘burnout,’ please check one” and rate their level of burnout from 1 (I enjoy my work. I have no symptoms of burn-out) to 5 (I feel completely burned out and often wonder if I can go on) [50]. Finally, lead clinicians are asked to “Rate your staff morale in your clinic” from 1 (*Poor)* to 5 (*Excellent*) [50].

### Practice characteristics

To measure organizational readiness, we use the 24-item Organizational Change Recipients’ Beliefs Scale (OCRBS). Response options ranged from 1 (*Strongly disagree*) to 5 (*Strongly agree*) [39].

### Barriers and facilitators to implementation and sustainability

Qualitative interviews explore the implementation process and the potential for sustainability guided by CFIR. In a purposive sample of eight sites per wave, two staff members per site are selected to participate in an interview six months after the end of the intervention. The interviews explore specific elements of the transformation processes to achieve TBC and HTN management and outcome goals, team member experiences with TBC, how various challenges were identified and addressed, satisfaction with the PF process, and strategies for sustaining gains.

In addition, qualitative interviews will be conducted with the practice facilitators at 12 months to further assess the implementation process, including contextual facilitation barriers, and facilitators that influenced TBC implementation, review adaptations to components and of TBC, what worked/did not work, and recommendations for enhancing sustainability and scale up. Informed consent will be obtained from all subjects. All study participants will receive an email describing the study with a link to the survey; the landing page will include the consent form, and clicking the link will indicate consent. Lead clinicians and other staff members invited to participate in qualitative interviews will be asked to provide verbal consent. Those who complete the interview will receive a $50 gift card as compensation. The study protocol was reviewed and approved by the New York University Institutional Review Board.

### Analysis

#### Quantitative analysis

The primary outcome is the overall average score on the TBC assessment tool. The secondary outcome is patient BP control. The analysis of the effect of PF on the TBC adoption outcome measure will be based on a linear mixed model. Specifically, to assess the PF intervention effect compared to usual care, we will use a model with a random site effect, and fixed effects for time and study condition. Condition will be dummy coded with the control condition as a reference category. The fixed-effects coefficient for the contrast of the PF condition with the control condition will indicate the direction and magnitude of the difference on the overall average TBC score. Inclusion of a fixed effect for time accounts for any general time trends. We also will explore whether the time coefficient should have a random effect to capture differences in time trends across practice sites. Confidence intervals (95%) will be reported to convey precision. In addition to the effects of time and condition, covariates (e.g., organizational readiness) will be considered for inclusion in the mixed-effects model to reduce the within-group variance, if the potential covariate is predictive of the outcome.

For the BP secondary outcome, a generalized linear mixed model will be used. The count of HTN patients with BP control (< 140/90) will be regressed on fixed effects for time and condition, the natural logarithm of the total number of HTN patients as an offset, and a random site effect. For the count outcome, a Poisson model will be used, but we also will consider a negative binomial mixed model if overdispersion is found. The offset term allows us to model the *rate* of BP control in each practice site and adjusts for differences in the number of HTN patients across sites. Comparisons of conditions (e.g., PF vs. control) can be converted to incidence rate ratios (IRRs) by exponentiating the appropriate fixed-effects coefficient or contrast.

Models will be fit using the *glmmTMB* package using R software [51, 52]. Each of the PF intervention effects will be tested using a two-sided level of significance α = 0.05. For the blood pressure secondary outcome, we will also conduct sub-analyses to assess outcomes by race/ethnicity.

Potential moderator effects will be examined by including separately each organizational characteristic as a main effect and in interaction with the treatment condition indicator (Control vs. PF vs. Sustainability). A significant interaction effect would indicate the effect of the condition depends on the moderator, and contrasts of the control and PF periods at different levels of the moderator would be undertaken to understand the pattern of the interaction. We will conduct further analysis to explore the possibility that changes in organizational readiness mediate the effect of PF on primary and secondary outcomes. We will conduct preliminary analyses to assess whether PF (versus Control) is associated with changes at the site level. If there is a detectable effect of the PF intervention on these measures, we will assess whether the changes in these measures are associated with changes in overall average TBC and HTN management scores and changes in the rate of BP control using the Baron and Kenny “product method” as a starting point of the analysis [53]. If necessary, we will explore more complex structural equation models (SEM) adapted for Poisson outcomes [54] as well as more recently developed causal inference methods [55, 56] to evaluate the mediation.

#### Power analysis

With the pre-specified number of sites (90) and design (Table 1; stepped-wedge design with 3 waves and 6 time periods; n = 432 TBC overall average scores), we investigated the PF intervention effect that is detectable with at least 80% power of a 2-sided significance test with α = 0.05. Power was calculated by simulating ten thousand datasets with modest correlation of TBC overall average scores over time (*r* = 0.35; ICC = 0.32) [57], and a standardized mean difference between control and PF conditions of *d* = 0.5 (i.e., a difference of half a standard deviation). Power was 0.92 to detect this effect with the planned number of sites. To allow for withdrawal of a small number of sites after baseline, we plan to enroll 90 practice sites (30 in each wave). Power for the secondary blood pressure outcome was also calculated by simulating ten thousand datasets. In all conditions, we assume an average of 150 patients with HTN per site and time period (i.e., 64,800 observations of patient BP over all sites and time periods). The intraclass correlation for BP control was specified as ICC = 0.85 based on an estimate from our previous EvidenceNOW study [25]. For the control condition, we specified a rate of BP control of 70%. For the PF condition, we specified an increase in the rate of BP control to 75%, and for the follow-up condition, we specified a decrease to 72%. The difference between the control and PF conditions corresponds to IRR = 1.07. Power to detect this IRR was > 0.99.

#### Fidelity analysis

We will calculate adherence to the PF protocol as the number of components fully or partially implemented divided by the total number of possible components [58]. We will assess visit dose (i.e., number of visits completed, type of visit conducted (in person vs. remote), and length of visit). We will compare pre and post-intervention HTN checklist responses to changes in the number of evidenced-based HTN strategies implemented at each practice.

#### Qualitative analysis

Interviews are transcribed verbatim and imported into Atlas.ti for analysis. The study uses an abductive content analysis that systematically integrates practices from inductive analysis (e.g., grounded theory) [59] and deductive analysis (i.e., applying codes associated with the guiding framework [CFIR]), while remaining open to informative deviations from those frameworks. Coding begins with an independent reading of the transcripts to identify preliminary themes, relevant patterns, and generative questions, followed by focused coding to identify clustered concepts, organize ideas, identify major emergent themes, and link them to relevant theoretical constructs. Throughout, coders will meet to review their coding, conduct team debriefing meetings, and reach a consensus on code names and meanings [60]. Once all transcripts are collaboratively coded, analytic domains are identified, and major and minor thematic areas described. Two coders independently code at least 10 interviews to establish the inter-rater reliability (IRR) and if it is inadequate (Krippendorff’s alpha < 0.80) [61], the lead investigators will work collaboratively to refine and/or clarify the coding scheme and provide additional coder training. The research team refines and/or clarifies the coding scheme and provides additional coder training. Double coding continues until adequate IRR is achieved.

#### Data integration

The analysis integrates the qualitative and quantitative data in a nested, parallel mixed-methods design [62–64]. Each interview is linked to descriptors of the originating study site’s characteristics, including potential moderators, mediators, and outcomes. These descriptors are used during analysis to categorize and interpret qualitative data in Atlas.ti using group functions. We triangulate findings from the baseline assessment, 12, and 18, -month adoption and sustainability data waves by developing a thematic matrix that includes practice and team-level characteristics and compare side-by-side those factors that were identified as facilitating or hindering the introduction, adoption, and sustainability of TBC for HTN strategies. This analysis examines how themes emerging from the qualitative data converge with, contextualize, explain, or expand the quantitative findings.

## Discussion

PF is a promising strategy to support the effective implementation of TBC in SIPs, but is understudied in small practices that serve immigrant and minority populations that are experiencing significant disparities in CVD-related health outcomes [65–67]. This study begins to fill this gap by leveraging BEHS’ unique, large network of urban small practices to test the hypothesis that PF can increase TBC, even in the context of resource constraints, to improve HTN outcomes. Of note, the investigative team and BEHS are using a pragmatic design that is aligned with BEHS’ and practice’s preferences that all practices will receive the intervention over time. The study further addresses the lack of theory-driven analyses by applying organizational theory to explore the mechanisms by which PFs impact organizational readiness and adoption of TBC and clinical outcomes.

An additional strength of the study is the partnership between NYU investigators and the NYC Department of Health and Mental Health’s practice network, a group of practices that serve primarily, low-income, minority, and immigrant populations. Embedding research in their PF program, BEHS increases the potential for sustainable changes.

Potential limitations may include the potential for practices dropping out of the study, lack of fidelity due to intervention adaptions, and non-response bias. Additionally, we anticipate that facilitators may experience challenges in meeting with staff due to high patient volume or staffing turnover. However, the BEHS has long-standing relationships with their network practices and providers have noted the advantage of the PF process which facilitates engagement [18, 68]. If we encounter lower than expected responses, practice facilitators will conduct in-person and phone outreach to increase rates of engagement. Also, facilitators will receive training and ongoing supervision to support the intervention and engagement process. We are using a rigorous process to assess fidelity.

Despite the potential limitations, this study provides much-needed guidance on how to optimize the adoption and sustainability of TBC in independent primary care settings to reduce the burden of disease related to suboptimal BP control and address current gaps in prior research [20].

## Supplementary Information

**Additional file 1.**

## Data Availability

The datasets generated and/or analyzed during the current study, including the PF Toolkit and all survey instruments, are not publicly available because of its use of information regarding private business operations, but will be made from the corresponding author on reasonable request. The details of data management procedures will also be made available by the corresponding author.

## Abbreviations

BEHS: Bureau of Equitable Health Systems
BP: Blood Pressure
CBPR: Community-Based Participatory Research
CFIR: Consolidated Framework for Implementation Research
CVD: Cardiovascular Disease
DOHMH: Department of Health and Mental Hygiene
EHR: Electronic Health Record
FTE: Full-time Equivalent
NCQA: National Committee for Quality Assurance
HHTeams: Healthy Hearts Teams
HTN: Hypertension
IRR: Inter-Rater Reliability
MOU: Memorandum of Understanding
OCRBS: Organizational Change Recipients’ Beliefs Scale
PCTGA: Primary Care Team Guide Assessment
PDSA: Plan-Do-Study-Act
PF: Practice Facilitation
QA: Quality Assurance
QI: Quality Improvement
QC: Quality Control
RCA: Root Cause Analysis
RCT: Randomized Controlled Trial
REACH: Regional Electronic Adoption Center for Health
SEM: Structural Equation Models
SIPs: Small Independent Primary Care Practices
TBC: Team-Based Care
